# Functional biomarkers for chronic periodontitis and insights into the roles of *Prevotella nigrescens* and *Fusobacterium nucleatum*; a metatranscriptome analysis

**DOI:** 10.1038/npjbiofilms.2015.17

**Published:** 2015-09-23

**Authors:** Szymon P Szafrański, Zhi-Luo Deng, Jürgen Tomasch, Michael Jarek, Sabin Bhuju, Christa Meisinger, Jan Kühnisch, Helena Sztajer, Irene Wagner-Döbler

**Affiliations:** 1 Research Group Microbial Communication, Department of Molecular Infection Biology, Helmholtz Centre for Infection Research (HZI), Braunschweig, Germany; 2 Genome Analytics, Helmholtz Centre for Infection Research, Braunschweig, Germany; 3 Institute of Epidemiology II, Helmholtz Zentrum München, German Research Center for Environmental Health (GmbH), Neuherberg, Germany; 4 Department of Conservative Dentistry, Ludwig-Maximilians-University, München, Germany

## Abstract

**Background/Objectives:**

Periodontitis is the most prevalent inflammatory disease worldwide and is caused by a dysbiotic subgingival biofilm. Here we used metatranscriptomics to determine the functional shift from health to periodontitis, the response of individual species to dysbiosis and to discover biomarkers.

**Methods:**

Sixteen individuals were studied, from which six were diagnosed with chronic periodontitis. Illumina sequencing of the total messenger RNA (mRNA) yielded ~42 million reads per sample. A total of 324 human oral taxon phylotypes and 366,055 open reading frames from the HOMD database reference genomes were detected.

**Results:**

The transcriptionally active community shifted from Bacilli and Actinobacteria in health to Bacteroidia, Deltaproteobacteria, Spirochaetes and Synergistetes in periodontitis. Clusters of orthologous groups (COGs) related to carbohydrate transport and catabolism dominated in health, whereas protein degradation and amino acid catabolism dominated in disease. The LEfSe, random forest and support vector machine methods were applied to the 2,000 most highly expressed genes and discovered the three best functional biomarkers, namely haem binding protein HmuY from *Porphyromonas gingivalis*, flagellar filament core protein FlaB3 from *Treponema denticola*, and repeat protein of unknown function from *Filifactor alocis.* They predicted the diagnosis correctly for 14 from 16 individuals, and when applied to an independent study misclassified one out of six subjects only. *Prevotella nigrescens* shifted from commensalism to virulence by upregulating the expression of metalloproteases and the haem transporter. Expression of genes for the synthesis of the cytotoxic short-chain fatty acid butyrate was observed by *Fusobacterium nucleatum* under all conditions. Four additional species contributed to butyrate synthesis in periodontitis and they used an additional pathway.

**Conclusion:**

Gene biomarkers of periodontitis are highly predictive. The pro-inflammatory role of *F. nucelatum* is not related to butyrate synthesis.

## Introduction

The microbiome of the oral cavity is the second most diverse one of the human body after the gut.^1^ Both have a profound influence on human health and their homeostasis can be influenced by toxins, diet, genetic factors and lifestyle, among others, potentially leading to dysbiotic communities and negative disease outcomes.^2^ Understanding the mechanisms leading to such ecological catastrophes is challenging in view of the enormous complexity of the microbiomes.^3^ However, the oral cavity is much more accessible than the gut, and just like many gut-related disorders, periodontitis results from a complex interaction between the microbiota and the host immune system. Therefore, studies of the role of the microbiota in periodontitis can not only help understand a potentially dangerous polymicrobial biofilm disease, but might reveal mechanisms that have a much broader relevance.

Periodontitis is a chronic inflammation of the gum that causes destruction of the alveolar bone and its final stage tooth loss. Globally, chronic periodontitis affects more than 10% of the human population^4^ and it is linked with systemic illnesses like cardiovascular disease and diabetes.^5,6^ It is a polymicrobial disease which results from the interplay between the subgingival biofilm and the host immune system.^7^ A checkerboard DNA–DNA hybridisation study of more than 13,000 plaque samples identified the so-called ‘red complex’ bacteria *Porphyromonas gingivalis*, *Tannerella forsythia* and *Treponema denticola* to be highly correlated with the clinical measures of periodontitis.^8^ Virulence factors of these species were studied in great detail and especially the role of *P. gingivalis* as a ‘key-stone’ pathogen has been well established.^9^ Next generation sequencing techniques have now described the bacterial communities in great depth and thus shifts in the abundances of many species were observed in periodontal disease.^10–12^ The identification of numerous additional species associated with disease resulted in the ‘polymicrobial synergy and dysbiosis’ model where synergistic activities of the whole community provoked by key-stone pathogens interfere with host immune defence and cause tissue destruction.^13^

Metagenomic studies are important to reveal the genetic potential of bacterial communities. However, microbes in the oral cavity are adapted to fluctuating environments and have multiple ways of generating energy and interacting with one another. This is reflected in the functional redundancy of their genomes, and therefore the actual activities of the microbes *in vivo* cannot be deduced from metagenome surveys. In the polymicrobial biofilm, the activity of individual species is strongly influenced by other microbes in the community. For example, the pathogenicity of *Streptococcus mutans* was switched on by co-cultivation with *Candida albicans*; moreover, those two periodontal pathogens utilised the medium much more efficiently when cultivated together *in vitro*.^14^ The exact growth conditions in the inflamed periodontal pocket, e.g., oxygen concentration, host proteins, microbial metabolites, etc. are largely unknown and are also expected to profoundly influence the activity of the microbes. As cultivation-based studies are hampered by the artificial conditions that need to be applied and by the sheer number of species interactions that would have to be studied, analysing the total messenger RNA (mRNA) of the periodontal pocket microbial communities allows for the first time to observe the transcriptional activity of the community as a whole under *in vivo* conditions. Only few such studies have yet been conducted, but they already provided important information. Duran-Pinedo *et al.* ^15^ showed that expression of genes for iron acquisition, synthesis of lipid A and flagella synthesis was increased in periodontitis. Interestingly, the authors found that the majority of virulence factors upregulated in disease originated from organisms which are not considered major periodontal pathogens. Thus, so-called ‘commensals’ were turned into pathogens in the dysbiotic community, strongly supporting the polymicrobial synergy and dysbiosis model. This was confirmed in a study of disease progression, where changes in the metatrascriptome between stable and progressive periodontitis sites were compared; again some of the health-associated bacteria were highly active in transcribing virulence factors.^16^ Jorth *et al*.^17^ studied the periodontal communities of aggressive periodontitis and found that community composition varied between patients, and gene expression also varied between patients, but functional profiles were conserved; specifically, pathways for fermentation of lysine to butyrate, histidine catabolism, nucleotide biosynthesis and pyruvate fermentation were upregulated in aggressive periodontitis. The authors hypothesised that *Fusobacterium nucleatum* might be a new key-stone periodontal pathogen which promotes disease by producing butyrate.

It is a big challenge to extract biologically meaningful information from the wealth of omics data currently generated and to translate it into clinically useful knowledge and tools. One approach is the search for biomarkers. Functional biomarkers might allow following disease progression and investigate the question of ‘hen and egg’. In the context of individualised medicine, they might detect shifts in the periodontal community before clinical symptoms are observed, which are generally unsensitive and late in disease progression, and therefore intervention could be early and much more successful. Such biomarkers are not available. Current potential biomarkers for periodontitis rely on general markers of the host inflammatory response; however, the presence of several periodontal pathogens, particularly *P. gingivalis*, or their proteins in saliva has been shown to be correlated with periodontitis.^18,19^

Here we studied the periodontal metatranscriptomes of 16 individuals, 6 of which had been diagnosed with chronic periodontitis. We show clear disease- and health-associated functional profiles and narrow down the changes in the gene expression profiles to three highly discriminative functional biomarker genes. Metatranscriptome studies differ widely in the applied methods, from sampling over preservation, RNA extraction, RNA amplification and sequencing method to bioinformatics analysis.^15–17^ Therefore, we used the raw data from the patients investigated by Jorth *et al.,*^17^ which were obtained in a completely independent study to validate our biomarkers. Finally, we took a closer look at the mechanisms shaping metatranscriptome patterns. We compared gene expression of the commensal *Pr. nigrescens* in health and disease and analysed in depth the pathways and microbial species that contributed to butyrate synthesis, challenging the previous hypothesis on the possible role of *F. nucleatum*.

## Materials and Methods

### Participants and ethics statement

The present study was part of the pre-test 1 phase of the ‘German National Cohort’.^20^ The German National Cohort is an epidemiological study that will recruit 200,000 individuals (aged 20–69) and monitor their health for a period of 30 years to study the development of chronic diseases and to develop strategies for their early detection and prevention. In the pre-test 1 phase, the feasibility of methods that might be used later in the main study was tested. Here we studied periodontal metatranscriptomes of the same individuals whose periodontal community composition had been determined previously by 16S ribosomal RNA (rRNA) gene amplicon sequencing.^12^ The study protocol was approved by the local ethics committee (Bayerische Landesärtzekammer, Munich, Germany), and written consent was obtained from all the participants. Average demographic and clinical characteristics of the studied individuals are shown in Supplementary Table S1 and data on each individual participant are provided in Supplementary Table S2.

### Clinical examination and sample collection

Clinical examination and sample collection were performed as described,^12^ except that paper points were incubated in RNAprotect (Qiagen, Hilden, Germany) for 5 min at room temperature immediately after sampling and subsequently stored at −80 °C until RNA extraction. Two paper points were sampled per site and multiple sites were sampled in one individual. From each pair of paper points, one was used for the RNA isolation (this study) and one for the DNA extraction.^12^ Paper points originating from the same individual were pooled together before RNA extraction.

### RNA extraction and mRNA sequencing

Chemicals were obtained from Sigma-Aldrich (Sigma-Aldrich, Taufkirchen, Germany) and the kits were used according to the manufacturer’s instructions, if not stated otherwise. All glassware and other instruments were RNase-decontaminated using RNase ZAP solution (Ambion, Austin, TX, USA). The paper points were thawed and shredded with sterile scissors. The fragmented paper points were incubated in lysis buffer containing 10 mM Tris, 1 mM EDTA, pH 8.0, 2.5 mg/ml lysozyme and 50 U/ml mutanolysin at 25 °C for 1.5 h on a shaking incubator at 350 r.p.m. A total 700 μl of fresh buffer RLT (Qiagen) containing 1% (v/v) β-mercaptoethanol was added and vortexed for 10 s. Samples (including the fragmented paper points) were placed on a QIAshredder Mini Spin column (Qiagen) and centrifuged for 1 min at 11,000 r.p.m. The flow-through containing the bacterial cells was mixed with 150 mg acid-washed and autoclaved glass beads (diameter 106 μm). Samples were vortexed 10 times for 30 s at full speed with at least 1-min intervals on ice in between vortexing and then centrifuged for 1 min at maximal speed. Total RNA was isolated from the supernatants with the RNeasy Mini Kit (Qiagen). DNA was removed by column digestion and by DNAse digestion in the eluate using the RNeasy cleanup procedure. Human and bacterial ribosomal RNAs were depleted using the MicrobEnrich and MicrobExpress kits (Ambion). The quality and quantity of RNA was assessed using the Agilent 2100 Bioanalyzer (Agilent Technologies, Palo Alto, CA, USA). The enriched mRNA was converted to cDNA libraries using the TruSeq RNA Sample Preparation Kit (samples 2, 3, 5 and 6; Epicentre Biotechnologies, Madison, WI, USA) and ScriptSeq, version 2, RNA-Seq Library Preparation Kit (other samples; Epicentre Biotechnologies). The quality of the libraries was assessed using the Agilent 2100 Bioanalyzer (Agilent Technologies). Three samples (nos 3, 4 and 6) were lost during RNA extraction, mRNA enrichment and cDNA library synthesis. For sequencing, equal amounts (12 pM) of the libraries were multiplexed. Cluster generation was performed with cBot (Illumina, Inc., San Diego, CA, USA) using TruSeq SR Cluster Kit version 3—cBot-HS (Illumina). Eight libraries with different indices were pooled and sequenced paired-end in the rapid mode in a single lane on the Illumina HiSeq 2500 using TruSeq SBS Kit version 3—HS (Illumina) for 110 cycles. A total of two lanes were sequenced for the 16 libraries. Image analysis and base calling were performed using the Illumina pipeline, version 1.8. RNA-seq sequencing data are available at MG-RAST under Project accession number 5148 (http://metagenomics.anl.gov/linkin.cgi?project=5148). After quality control, ~42 million reads were obtained per sample. After removal of ribosomal RNA, on average ∼5 million reads per sample could be assigned to open reading frames (ORFs) by the MG-RAST pipeline (Supplementary Table S3).

### Data processing

The sequencing output was quality controlled and the sequencing adaptors were clipped using the fastq-mcf tool of ea-utils.^21^ Sequence reads were analysed with an in-house pipeline and MG-RAST.^22^ The in-house pipeline started with the removal of ribosomal RNA reads using SortmeRNA version 1.8.^23^ Non-ribosomal RNA fragments were mapped against the HOMD reference sequences (the 397 annotated genomes) using the Burrows–Wheeler Aligner version 0.7.5 (−k 31, option for minimum seed length, SA (SA:Z), tag for chimera removal), and SAMtools for sorting and filtering nucleotide sequence alignments.^24^ Merging the paired reads after calculating hits per ORF and genome was performed using custom user scripts. Differential gene expression was calculated using the R package edgeR.^25^ Using the MG-RAST server, genes were assigned to COG (Clusters of Orthologous Groups) categories according to MG-RAST Hierarchical Classification (maximum e-value e-5, minimum identity 60%, minimum alignment length of 15 amino acids). The COG database from 2003 was used.^26^

### Statistical analysis

R, the free software environment for statistical computing and graphics^27^ and PRIMER and PERMANOVA+, the suite of univariate, graphical and multivariate routines^28^ were used to analyse the data. Rarefaction curves were plotted on unstandardised mapped reads (raw counts) grouped to features, e.g., COGs, using the R package vegan.^29^ A principal coordinates analysis (PCoA) was performed with the PCO PERMANOVA+ routine on the basis of the Bray–Curtis similarity matrix created for the standardised log-transformed abundances of reads grouped to classes, COGs or genes. Scatter plots of the first two principal coordinates were generated and in the case of class-abundances-based PCoA plots, a vector overlay was used to visualise the relationship between the abundances of taxonomic classes and the ordination axes. Each vector begins at the centre of a circle (0, 0) and ends at the coordinates (*x*, *y*) consisting of the Spearman's rank correlation coefficient between that variable and each of the ordination axes 1 and 2, respectively. The length and direction of the vector indicate the strength and direction, respectively, of the relationship between the variable and the ordination axes. Group-average agglomerative hierarchical clustering was performed on the basis of the Bray–Curtis similarity matrix. A heat map was created using the R package pheatmap.^30^ The linear discriminant analysis (LDA) effect size (LEfSe) pipeline^31^ was used to identify features (i.e., phylotypes, COGs or genes) that were associated with health and disease. This software is available at (http://huttenhower.sph.harvard.edu/galaxy/). Samples 1 and 11 were excluded from all the analyses that identified features associated with periodontal disease and health.

To select the best gene markers, two feature selection techniques which are integrated in the Python package scikit-learn^32^ were applied. They were: (i) recursive feature elimination and (ii) random forest (RF) feature importance evaluation method. The recursive feature elimination is a widely applied algorithm in feature selection because of its high performance. It recursively eliminates the features with low weights measured by an external classifier such as support vector machine (SVM) until a set of best features are achieved. RF is a supervised machine learning method generally used in classification, regression and feature importance evaluation. RF constructs the decision trees with different subsets of features and thus enables the evaluation of the importance of each feature. The standardised abundance of the 100 genes with the highest LDA score was scaled into the range of 0 to 1 and afterwards applied to the feature selection process. To validate the discovered biomarker candidates, a model was built for predicting the diagnosis of individuals from the training data set using the SVM algorithm with linear kernel^33^ under the optimisation of fourfold cross validation. The optimal c parameter of the SVM model was determined by a grid search in the range: [2^−5^, 2^7^]. An external data set^17^ was used for further validation. Computer code used to generate results can be accessed at (https://github.com/dawnmy/metatranscriptome_paper).

## Results

Our data set comprises 666 million raw reads originating from 16 periodontal communities (Supplementary Table S3). After quality control, 616 million of reads remained. Using SortMeRNA, 19% of them were identified as eukaryotic rRNA and 60% as bacterial and archaeal rRNA. Using HOMD and Burrows–Wheeler Aligner, 4% chimeras were detected and the remaining 17% were putative mRNA of both bacterial and eukaryotic origin. Of those 104 million reads, 27% could be mapped to the 397 genomes in the HOMD database, on average 1,758,071 reads per sample. Independent Burrows–Wheeler Aligner mapping of the quality controlled raw reads to the human genome showed that 18% of them were of human origin.

For the COG analysis, we used the MG-RAST server which applies different tools for quality control and for identifying ribosomal RNA. Using MG-RAST, 77.9% of quality controlled reads were identified as so-called artificial duplicate reads (ADR, mostly rRNA or sequencing artifacts), 5.3% were non-artificial duplicate reads and non-ORFs, and 16.8% were potential mRNA as they contained a predicted ORF. Of those 81 million reads, 8.8% could be annotated with COG categories.

### Phylogenetic composition of periodontal metatranscriptomes

**Figure 1:**
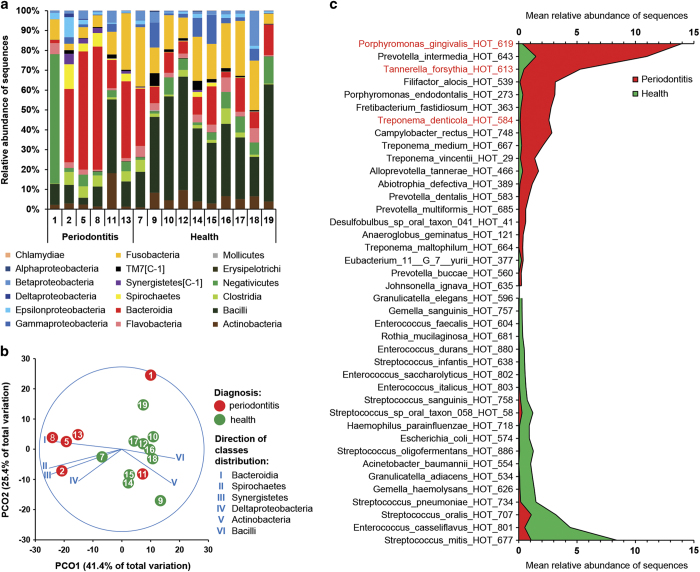
Composition of microbial communities from periodontal pockets as assessed by metatranscriptome analysis. (**a**) Composition of the periodontal community in the 16 individuals shown on the class level. Healthy subjects and those diagnosed with periodontitis are grouped on the right and left side of the subpanel, respectively. (**b**) Principal coordinates analysis (PCoA) of periodontal communities. Bray–Curtis similarity values were calculated on standardised log_2_ transformed abundances of reads grouped to Classes. Communities from healthy individuals and those with periodontitis are marked in green and red, respectively. Vectors represent the direction of the relationships between classes and ordination axes (see Materials and Methods for details). (**c**) Transcriptionally active species associated with health and disease. Relative abundance in health and disease of reads grouped to species (human oral taxons, HOTs) that were associated with periodontitis (upper part) and health (lower part). ‘Red-complex’ species are highlighted in red. *P*<0.05 for all data shown.

Figure 1a shows the assignment of the mRNA reads to phylogenetic classes. Communities 2, 5, 8 and 13 from periodontitis patients were dominated by Bacteroidia transcripts. Transcripts from Spirochaetes and Synergistetes were less abundant but found almost exlusively in those samples. Periodontitis samples 1 and 11 showed strikingly different profiles. Sample 1 was dominated by Negativicutes, while sample 11 was dominated by Bacilli. Transcripts from Actinobacteria were more abundant in ‘healthy’ samples (with the exception of the discordant community 11). Transcripts from several phyla that were present according to 16S amplicon sequencing were missing: (SR1 [C-1], Chloroflexi [C-1], Sphingobacteria, Bacteroidetes [C-2] and Bacteroidetes [C-1]). Those classes had abundances below 0.5% in the taxonomic profiles based on amplicon sequencing^12^ and thus their contribution to the metatranscriptome might be too small to be detectable.

The PCoA distinguished communities from individuals diagnosed with periodontitis from those that were healthy (Figure 1b). The classes Bacteroidia, Deltaproteobacteria, Spirochaetes and Synergistetes showed a strong negative correlation with the principal coordinate 1 in contrast to the classes Actinobacteria and Bacilli that were positively correlated. This demonstrates a shift in the active community from health, where an aerobic Gram-positive flora is dominant, and disease where anaerobic Gram-negative bacteria are more prevalent. Samples 1 and 11 had previously been identified as outliers by 16S rRNA gene amplicon sequencing.^12^

### Most active bacterial taxa in the periodontal microbiome

Using the LEfSe algorithm, we compared the transcriptional activity of periodontal bacteria in health and disease. Reads were mapped to the ORFs in the reference genomes of oral bacteria and the percentage of transcripts from each taxon was calculated for each community. Transcripts were grouped to species-level taxa named human oral taxon, which includes species that have not yet been cultivated or validly described. Transcripts from a total of 324 human oral taxon phylotypes were identified. Of those, 76 were associated with health and 33 with periodontitis (LDA >2, *P*<0.05, Supplementary Datasheet S1).

In Figure 1c, the mean abundances of transcripts from the 20 most active taxa are shown. In periodontitis, the ‘Red-complex’ bacteria *P. gingivalis*, *T. forsythia* and *T. denticola* were among them, as well as other known periodontal pathogens like *Pr. intermedia*, *Filifactor alocis*, *P. endodentalis*, *Fretibacterium fastidiosum*, *Campylobacter rectus* and several species of *Treponema.* These taxa have previously already been associated with periodontitis at the DNA level using 16S rRNA gene amplicon sequencing for the same set of samples,^12^ and here we further confirmed it at the mRNA level. Thus these species are not only abundant, but they also belong to the most active part of the community in periodontitis.

The healthy metatranscriptomes were dominated by transcripts from streptococci, represented by *S. mitis*, *S. oralis*, *S. pneumoniae*, *Streptococcus sp.* (human oral taxon 58), *S. sanguinis* and *S. infantis*.

### Functional shifts in the dysbiotic periodontal community

In the 16 studied communities, we were able to assign 7.65 million reads to 4,256 different COGs. Each COG consists of clusters of orthologues and is assumed to have evolved from a single ancestral gene.^26^ All communities except sample no. 9 reached a clear plateau according to the rarefaction analysis (Supplementary Figure S1a). Because 42% of the COGs had a low abundance (below 0.01% in any community) and together contributed only 0.9%±0.4 (s.d.) of the total abundance, they were excluded from further analyses. PCoA (Supplementary Figure S1b) indicated that the COG composition of the metatranscriptomes was quite similar for all communities except for sample 9 that was an outlier, likely owing to the low sequencing depth. Periodontitis samples 2, 5, 8 and 13 tended to group in the top right area of the plot but there was no clear border between them and samples from healthy individuals.

**Figure 2:**
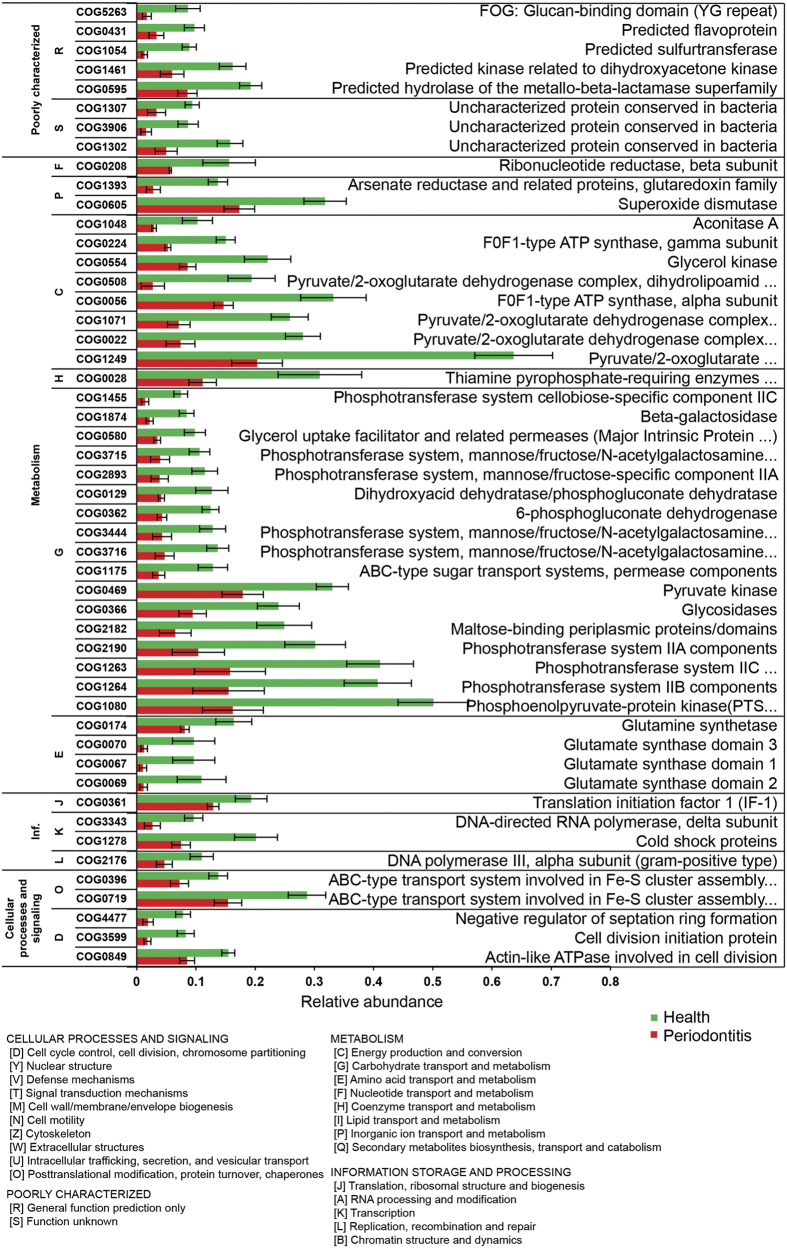
COGs associated with health. Relative abundance in health and disease of reads grouped to the 50 COGs with the highest LDA score which were associated with health. The error bars show the standard error of the mean. *P*<0.05 for all COGs shown. COG, cluster of orthologous groups; Inf., information storage and processing; LDA, linear discriminant analysis.

Clear differences between periodontitis and health were found using the LEfSe algorithm. A total of 334 COGs were significantly differentially expressed (Supplementary Datasheet S2). Of those, 127 COGs were more abundant in health and 207 were more abundant in disease (*P*<0.05, LDA score >2). The 50 COGs with the highest LDA scores in health are shown in Figure 2. Pyruvate/2-oxoglutarate dehydrogenase complex was the most strongly enriched health-related COG in the whole data set, followed by phosphoenolpyruvate-protein kinase. The health-associated COGs related to carbohydrate transport and metabolism (category G) included many different phosphotransferase systems, ABC-type transport systems, permeases and hydrolysing enzymes like beta-galactosidase and glycosidase, as well as 6-phosphogluconate dehydrogenase, an enzyme in the pentose phosphate pathway, and pyruvate kinase involved in glycolysis. Other COGS involved in energy production and conversion (category C) were aconitase catalysing the isomerisation of citrate to isocitrate, F_0_F_1_-type ATP synthase and glycerol kinase. Glutamate synthase and glutamine synthetase transcripts were also enriched in communities from healthy individuals, as well as the stress-related superoxide dismutase and cold shock proteins. The data suggest that in the healthy periodontal microbiome uptake of dietary sugars and their metabolism in central pathways predominates, cell division is rapid, and oxidative stress needs to be counteracted. The enrichment for glutamine synthetase and glutamate synthase transcripts might indicate a need to synthesise this essential amino acid.

**Figure 3:**
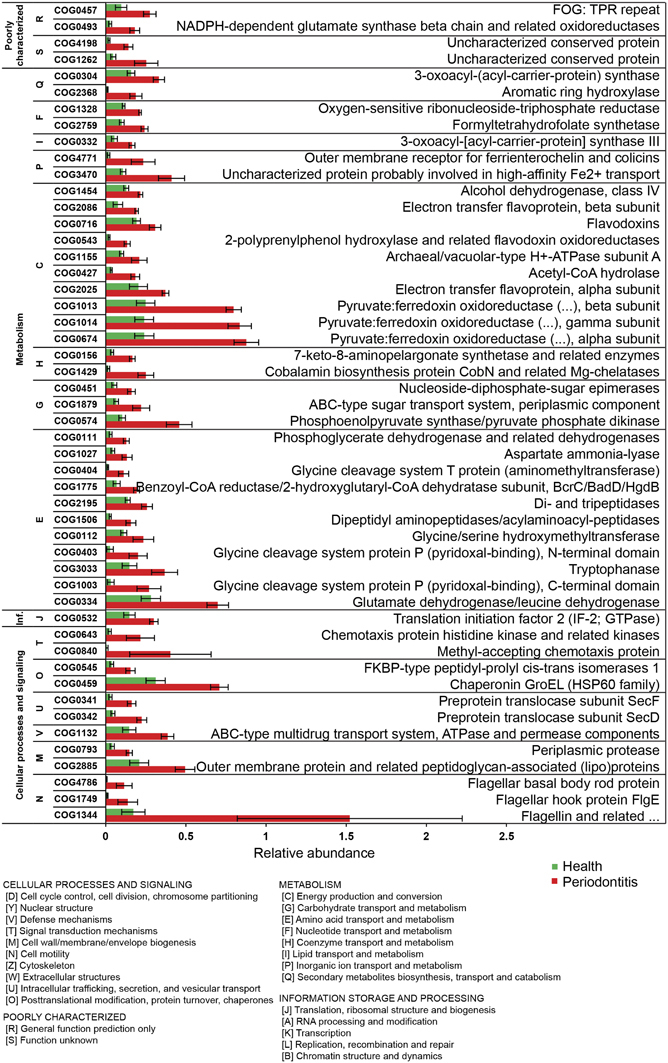
COGs associated with periodontitis. Relative abundance in health and disease of reads grouped to the 50 COGs with the highest LDA score that were associated with periodontitis. The error bars show the standard error of the mean. *P*<0.05 for all COGs shown. COG, cluster of orthologous groups; Inf., information storage and processing; LDA, linear discriminant analysis.

In the periodontitis metatranscriptomes (Figure 3), a number of enriched COGs can directly be linked to pathogenesis. The most abundant one was COG1344 (flagellin), but additionally transcripts assigned to chemotaxis (category T), iron acquisition, antimicrobial resistance, secretion (category U and V), colicin receptor and Fe transport protein (category Q) were significantly more abundant. Community metabolism (categories E, G, H, C, I, F, Q) was different in periodontitis and was characterised by the enrichment of COGs related to peptide catabolism (dipeptidyl aminopeptidases, dipeptidases and tripeptidases) and degradation of amino acids (category E, aspartate, glycine, glutamate, leucine, tryptophan). This finding is in accordance with the known high concentrations of peptides and amino acids in periodontal pockets. They originate from host serum or damaged tissues as a result of the strong proteolytic activity of the microbial communities as well as destructive immune response.^34^ The glycine cleavage system (also known as glycine decarboxylase complex or glycine synthase) carries out the formation of serine from two glycine molecules and is composed of four proteins called T-, P-, L- and H-protein. COGs for the P-, T- and H-protein were significantly more abundant in periodontitis. From serine, pyruvate is formed by the serine dehydratase and can then be metabolised by the pyruvate–ferredoxin oxidoreductase. COGs for the alpha, beta and gamma subunits of this enzyme were the most strongly differentially expressed COGs in the periodontitis metatranscriptomes (after flagellin). In addition, flavodoxins (COG0716) and flavodoxin oxidoreductases (COG0543) were enriched. Flavodoxin, like ferredoxin, is a low-potential electron carrier, but in contrast to ferredoxin, it does not contain iron; flavin mononucleotide functions as redox group. Therefore, flavodoxin is synthesised by anaerobes under iron-limiting conditions. The enrichment of COG3470 (uncharacterised high affinity Fe^2+^ transport protein) further suggests that iron is limited in disease.

Thus, based on the analysis of transcripts grouped to COGs, we observed a shift from the carbohydrate-consuming healthy subgingival flora to the dysbiotic periodontitis community that relied on proteolysis and fermentative pathways and was limited for iron. Moreover, known virulence factors (chemotaxis and flagella genes) were enriched.

### Gene biomarkers for periodontitis

We mapped 28.1 million reads to 366,000 genes from reference genomes of oral microorganisms. A cluster analysis of the 5,000 most highly expressed genes encompassing 40% of all mapped reads (Supplementary Figure S2) showed clear differences in the expression profiles of the microbial communities in health and periodontitis. The clustering was not changed if the number of genes analysed was increased to 80% of the most highly expressed genes (Supplementary Figure S3). Interestingly, sample no. 7 (health) clearly clustered with the periodontitis samples. It would be interesting to test whether such a disease-like expression profile in a clinically healthy individual indicates an early stage of periodontitis. Conversely, sample no. 11 clustered with the ‘healthy’ samples, although it was derived from an individual diagnosed with periodontitis. This community was dominated by health-related *Rothia* sp. and streptococci, but we also detected weak activity of *P. gingivalis*, *Pr. intermedia* and *T. forsythia* (Supplementary Figure S4). The periodontal pocket depth in this individual was relatively shallow (4–5 mm), similar to the other outlier (sample no. 1).This may suggest that in individual 11 we observed a mild form of periodontal disease, e.g., gingivitis.

We used the LEfSe algorithm to detect gene biomarkers. The standardised abundances of reads from the top 2,000 most highly expressed ORFs were used for this calculation, and samples 1 and 11 were excluded. Of those 2,000 most highly expressed ORFs, 420 were significantly associated with periodontitis and 329 with health (*P*<0.05; Supplementary Datasheet S3). Among the top 100 marker genes, the majority (48) originated from *P. gingivalis*, followed by 16 from *Pr. intermedia*, 10 from *Fretibacterium fastidiosum*, 9 from *Filifactor alocis*, 7 from *Tr. denticola*, 5 from *Ta. forsythia* and 5 from the other species. Marker genes included genes encoding structural proteins, metabolic enzymes and numerous virulence factors. Among the best markers, we identified both genes encoding the arginine-specific gingipains (*rgpA* and *rgpB*) and one coding for the lysine-specific gingipain Kgp. Gingipains, the cysteine proteases produced by *P. gingivalis*, are responsible for most of the proteolytic activity of this bacterium and are known virulence factors. Other potential biomarkers for periodontitis were *fimA* encoding fimbrillin, *hagA*, *hagE* encoding hemagglutinin proteins and *hmuY* encoding a potential haem transporter. Among marker genes that were involved in iron uptake, we also identified an iron transporter from *Campylobacter rectus* and a hemin-binding protein from *Pr. intermedia*. We also detected numerous genes related to flagella synthesis, e.g., flagellin from *Fretibacterium fastidiosum* and numerous flagella genes from *T. denticola*.

**Figure 4:**
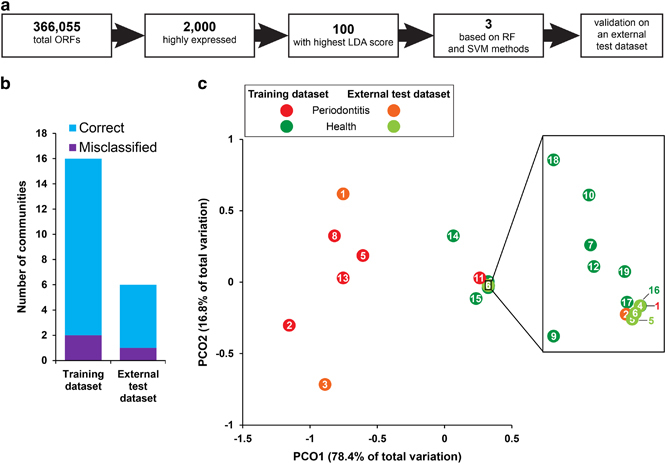
The biomarker candidates, their discovery and validation. (**a**) Flowchart for the biomarker selection and validation procedure. Reads were mapped against the HOMD database. LDA scores were calculated with linear discriminant analysis (LDA) for the 2,000 most highly expressed ORFs using the LEfSe algorithm.^31^ The top 100 ORFs with the highest LDA scores were further evaluated using the RF and SVM methods leading to the discovery of the three best biomarkers. They were validated using data from an independent study published previously. (**b**) Comparison between prediction based on the three biomarkers and clinical diagnosis. The classification model was tested on 22 samples. The 16 training samples are from our own study; the six external samples are from ref. 17. All the training samples were correctly classified except for two outliers. From the external data set, all but one sample were correctly classified. (**c**) Principal components analysis (PCA) was based on the three biomarker candidates. Euclidean distances were calculated on standardised abundances of reads from the three biomarker genes and scaled into the range of 0 to 1. Part of the plot was enlarged to clearly show the positions of densely packed samples. ORF, open reading frame; RF, random forest; SVM, support vector machine.

The PCoA analysis of the periodontal communities using the 749 biomarker genes identified by the LEfSe algorithm showed two clearly separate clusters for health and periodontitis (Supplementary Figure S4a). Sample 1 was an outlier as identified previously. In the middle area, we found four communities that may represent an intermediate state between health and disease. Three of them came from healthy individuals (sample 15, 14, 7) and one from a subject diagnosed with periodontitis (11). These communities harboured intermediate levels of transcripts from health- and disease-related periodontal microorganisms (Supplementary Figure S4b). We hypothesise that these samples (25% of our samples) might represent gingivitis, a very common mild state of periodontal disease. Although the power of discrimination increases with the number of biomarkers, the problem of overfitting arises, i.e., in a large enough set of variables (e.g., a metatranscriptome), biomarkers will be detected by chance. From a practical point of view, the number of biomarkers should also be narrowed down to develop diagnosis tools. Finally, with a smaller number of biomarkers, model performance can be improved and deeper insight into the underlying mechanisms can be obtained.^35^ The biomarker selection and validation process applied here is depicted in Figure 4a. To select a subset of biomarkers but keep their discriminatory power, we applied recursive feature elimination and the RF feature importance evaluation method. The recursive feature elimination was implemented with SVM. Both methods identified the same three best biomarkers from the top 100 genes with the highest LDA score (Supplementary Datasheet S3). Based on them, a model was achieved which misclassified only samples 1 and 11 that had previously been identified as outliers^12^ (Figure 4b). Further validation on an external data set^17^ misclassified one out of six samples (Figure 4b). This was sample no. 2 from an individual with periodontitis, which is an outlier as shown in the PCoA analysis (Figure 4c). The RF was also ultilised to build the classification model which showed the same outcome as the SVM model (our samples 1 and 11 and the external sample 2 were misclassified).

The three highly expressed biomarker genes encoded haem binding protein HmuY from *P. gingivalis*, flagellar filament core protein FlaB3 from *T. denticola* and a repeat protein of unknown function from *Filifactor alocis*.

### Transcriptome of *Prevotella nigrescens* in health and disease

Key-stone periodontal pathogens are hypothesised to influence the pathogenic potential of other bacteria in the community. *Pr. nigrescens* can easily be isolated from infected teeth, but its pathogenicity is unclear.^36,37^ Here we compared the transcriptomes of *Pr. nigrescens* in three communities from healthy individuals (sample nos 10, 12 and 17) with the transcriptomes in communities from individuals with periodontal disease (sample nos 5, 8 and 13). In the six selected communities, *Pr. nigrescens* showed a mean abundance of 4.4%±2.3% (s.d.). Moreover, the selected communities had a clear health- or disease-associated gene expression profile according to our gene marker analysis (Figure 1 and Supplementary Figure S4).

On average, 147,000 reads were assigned to *Pr. nigrescens* per sample (maximum 320,000; minimum 30,000), and mapped to 2,068 genes, representing 94% of the genome. We found 48 differentially expressed genes (*P*<0.05; Table 1 and Supplementary Table S4). The majority of them were upregulated in disease and were related to virulence: peptidases (M16 and M26), a haem ABC transporter, a multidrug transporter, collagenase and hemagglutinin. We observed the upregulation of l-asparaginase, l-aspartate oxidase, fumarate hydratase, acyl-CoA synthetase and NAD-utilising dehydrogenase. Moreover, a strong increase in the expression of a putative operon (pnig_c_6_996—pnig_c_6_999) was observed. It consists of four genes coding for d-glycero-d-manno-heptose 1-phosphate kinase, phosphoheptose isomerase, hydrolase (likely d,d-heptose 1,7-bisphosphate phosphatase) and an uncharacterised protein. Glycero-manno-heptose is present in cell surface polysaccharides and glycoproteins. It can be found in lipopolysaccharide and its derivatives can also be found in capsules, O-antigens and in surface layer glycoproteins.^38^ Thus, it is likely that the immunogenic properties of *Pr. nigrescens* changed in the dysbiotic community, which may result in different interactions with the host immune system.

*Pr. nigrescens* produces a bacteriocin named nigrescin which is active against the ‘red complex’ periodontal pathogens.^39^ We detected its expression *in vivo* both in health and periodontitis, indicating that it may be necessary to ensure survival of *Pr. nigrescens* in the competitive multi-species biofilm of the periodontal pocket.

*Pr. nigrescens* possesses haemolytic and hemagglutinating activity.^37^ Strikingly, the haemolytic protein was downregulated, suggesting that in periodontitis, iron is obtained via another route. Recently a mutualistic co-operation between *P. gingivalis* and *Pr. intermedia* was demonstrated *in vitro*, whereby haem degradation was accomplished by the joint activity of the HmuY haemophore of *P. gingivalis* and the cysteine protease interpain A (InpA) of *Pr. intermedia*.^40^ The HmuY haemophore was one of the most highly expressed genes in our study. *Pr. intermedia* and *Pr. nigrescens* are very closely related and show similar physiologies. Thus, our data provide *in vivo* evidence for this finding.

### Expression of pathways leading to butyrate

**Figure 5:**
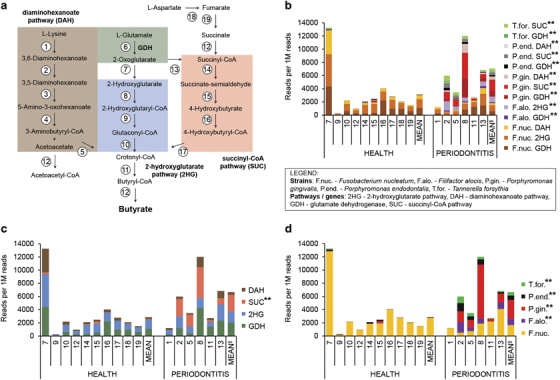
Pathways involved in butyrate production and their expression in health and periodontitis. (**a**) Scheme of the proposed pathways utilised by oral bacteria to produce butyrate from lysine, glutamate and aspartate: the diaminohexanoate pathway (DAH, highlighted in brown) of lysine degradation consists of the following enzymes (numbers within circles): (1) L-lysine-2,3-aminomutase, (2) L-β-lysine-5,6-aminomutase, (3) 3,5-diaminohexanoate dehydrogenase, (4) 3-keto-5-aminohexanoate cleavage enzyme, (5) 3-aminobutyryl-CoA deaminase. The glutamate dehydrogenase pathway (GDH, highlighted in green) catalyses the reaction number (6). The 2-hydroxyglutarate pathway (2HG, highlighted in blue) of glutamate catabolism consists of following enzymes: (7) 2-hydroxyglutarate dehydrogenase, (8) glutaconate (2-hydroxyglutarate) CoA-transferase, (9) 2-hydroxyglutaryl-CoA dehydratase, (10) glutaconyl-CoA decarboxylase, (11) butyryl-CoA dehydrogenase, (12) acyl-CoA:acetate CoA/acetoacetate CoA-transferase, (13) 2-oxoglutarate oxidoreductase. The succinyl-CoA pathway (SUC, highlighted in red) of glutamate catabolism consists of the following enzymes: (14) succinate-semialdehyde dehydrogenase, (15) 4-hydroxybutyrate dehydrogenase, (16) 4-hydroxybutyrate coenzyme A transferase, (17) 4-hydroxyphenylacetate-3-hydroxylase/4-hydroxybutyryl dehydratase. l-Aspartate can be metabolised to succinate by (18) and (19) that are aspartate aminotransferase and fumarate reductase, respectively. Succinate can enter the succinyl-CoA pathway (SUC). The presented pathways were adapted from refs 60–62. (**b**) Abundance of reads grouped to the six species and four pathways highlighted in **a**. The values are plotted on the left and right side for the healthy individuals and those with periodontitis, respectively. Mean values for both the groups are also shown. The legend with the list of abbreviations is located below the figure. (**c**) Same as **b** but reads are grouped to pathways only. Colours are in accordance with those used in **a**. (**d**) Same as **b** but reads are grouped to species only. Colours are in accordance with those used in **b**. **, showed statistically significant (*P*<0.01) higher abundance in the communities from individuals with periodontitis. The outlier sample nos 1 and 11 were excluded from the analysis.

Butyrate is a cytotoxic short-chain fatty acid^41^ produced by anaerobic bacterial metabolism and it is enhanced in chronic periodontitis;^42^ it can inhibit differentiation of human gingival fibroblast cells^43^ and the lysin degradation pathway leading to buryrate was shown to be upregulated in the metatranscriptome of aggressive periodontitis.^17^ Therefore, here we used our metatranscriptome data to analyse the expression of pathways leading to butyrate in detail. At least 10 oral bacterial species were reported to produce butyrate *in vitro*, and they utilise different amino acids and peptides through numerous pathways as summarised in Supplementary Table S5. Figure 5a shows those pathways in detail: lysine, glutamate and aspartate are catabolised to butyrate via the diaminohexanoate, 2-hydroxyglutarate and succinyl-CoA pathways as well as through the glutamate dehydrogenase pathway. The 19 enzymes that are performing the various transformations and the connections between the pathways are also indicated.

The abundance of reads coding for enzymes involved in those pathways and assigned to the various periodontal pathogens in our metatranscriptome samples is shown in Figure 5b (Supplementary Datasheet S4). The glutamate dehydrogenase, diaminohexanoate and 2-hydroxyglutarate pathways of *F. nucleatum* were universally expressed in all samples except sample no. 9, and reached an unusually high level in sample no. 7. Interestingly, in samples from individuals with periodontitis, additional species could be observed expressing various pathways leading to buryrate. Figures 5c and d show the abundance of the same reads as shown in Figure 5b but grouped to pathways only and species only, respectively. The diaminohexanoate, 2-hydroxyglutarate and glutamate dehydrogenase pathways were universally expressed, whereas the succinyl-CoA pathway was found almost exclusively in disease (Figure 5c). Similarly, butyrate pathways from *F. nucleatum* were universally expressed, whereas the pathways from *Tannerella forsythia*, *P.* spp. and *Filifactor alocis* contributed to butyrate production in disease only.

The data indicate that butyrate production by *F. nucleatum* is not specific for periodontitis. However, in the dysbiotic periodontal community, several additional pathogens contribute to butyrate production, and this community additionally utilises the succinyl-CoA pathway to catabolise glutamate. Thus, butyrate catabolism is performed by a taxonomically and functionally more diverse community in periodontitis.

## Discussion

We observed a clear shift in the transcriptionally active community between health and disease. Transcripts from Bacteroidia, Deltaproteobacteria, Spirochaetes and Synergistetes were highly abundant in disease in contrast to those from Bacilli and Actinobacteria that were enriched in health. A higher abundance of those four classes in periodontitis had previously been found using 16S rRNA gene amplicon sequencing of the same samples,^12^ indicating that both the abundance and the transcriptional activity of these bacteria were increased in periodontitis. The correlation between community fingerprints obtained with 16S profiling and metatranscriptomics was higher for periodontitis samples than for those from healthy individuals (*r*=0.84±0.13 vs. 0.68±0.23 (mean±s.d.; Supplementary Figure S5)). Similarly, Jorth *et al*.^17^ observed a higher correlation between rRNA abundance (RNA level) and rRNA gene abundance (DNA level) for samples from periodontitis than for those from healthy sites. This could be caused by technical problems. Deep pockets provide much more material than the shallow healthy sulcus, and thus it is more difficult to sample identical communities for DNA and RNA extraction from healthy individuals. Alternatively, the microbial community in chronic periodontitis may be more stable and therefore DNA and mRNA fingerprints are more similar in contrast to the healthy community, which is located closer to the rest of the oral cavity and may undergo dynamic changes. This hypothesis is in accordance with the lower diversity of the metatranscriptome in periodontitis,^17^ whereas DNA-based studies previously found a similar or higher alpha-diversity in disease.^10,12^ Here, we also observed that metatranscriptomes from diseased periodontal pockets were more similar than those from healthy subjects. Moreover, intermediate communities were also observed. In future studies with more samples, they could provide a hint towards the development of periodontitis and the causal role of the microorganisms.

To investigate functional changes in activity, we grouped sequence reads to COGs and identified those that were differentially expressed. At COG level 1 in periodontal disease, we observed a higher abundance of COGs related to protein hydrolysis, e.g., peptidases and enzymes for amino acid catabolism, and the glycine cleavage system. Higher glycine concentrations were detected in saliva from individuals with periodontitis^44^ and glycine is rapidly metabolised in a co-culture of *P. gingivalis* and *T. denticola in vitro*.^45^

The search for best potential biomarkers retrieved metabolic enzymes and numerous virulence factors like gingipains (multi-functional proteases secreted by *P. gingivalis* to manipulate the host immune system), hemagglutinins and fimbriae, most of which are in agreement with previous metatranscripome studies.^15–17^ This number was narrowed down using the RF and SVM methods to three biomarkers that predicted the diagnosis correctly for the training data set, i.e., all samples except for the two outliers identified previously.^12^ This had not been possible with the same samples using a combination of the 10 best phylogenetic biomarkers, e.g., 16S rRNA gene-based OTUs (operational taxonomic units).^12^ The performance of the phylogenetic biomarkers could not be improved by combining 50 or even 100 of them. Part of the reason may be that the phylogenetic diversity in periodontal pocket samples is much higher than the functional diversity. More importantly, the 16S rRNA gene is a phylogenetic marker with little information about functional traits of the organisms. It has been shown that two strains differeing in one nucleotide only can have widely different functional associations.^46^ Validation of the functional biomarkers with the metatranscriptome data from an independent study^17^ was successful for all but one sample, which according to the PCoA analysis was an outlier. This indicates that the method of sampling (paper points or curettes) and the details of RNA extraction, mRNA sequencing and bioinformatics analysis used in those two studies did not influence the results. This is also in contrast to taxonomic profiling of communities based on the 16S rRNA gene, which is strongly dependent on the primers and methods for OTU calling. Those three biomarkers will be discussed below.

### Haem binding protein HmuY, biomarker from *P. gingivalis*

Iron is an essential element for life because it is part of co-factors, cytochromes and iron–sulphur proteins. Thus, host and pathogen compete fiercely for iron using highly effective mechanisms for its sequestering and uptake.^47^ In vertebrates, haem is the most abundant iron source and therefore pathogens developed mechanisms for binding, uptake and degradation of haem.^48^ As a first step, haemoglobin must be proteolytically degraded. *P. gingivalis* uses so-called gingipains, arginine or lysine-specific proteases (Rgp, Kgp)^49^ which are also able to degrade cytokines thereby downregulating the host’s immune response.^50^ Transcripts coding for both proteases were among the 100 best biomarkers identified by LEfSe. However, a biomarker with an even better discriminative power was the haem binding protein HmuY which is highly specific for *P. gingivalis*.^51^ It has been shown *in vitro* that haem can be obtained cooperatively by the HmuY haemophore of *P. gingivalis* and the cysteine protease interpain A (InpA) from *Pr. intermedia*.^40^ This may also occur *in vivo*, as HmuY expression was correlated with a high expression of gingipains and, in some communities, with InpA.

### Flagellar filament core protein FlaB3, biomarker from *T. denticola*

In *T. denticola*, and more generally, in members of phylum Spirochaetes, flagella filaments are an integral part of the cytoskeleton, contributing to cell shape and enabling motility.^52^ Flagella filaments of *T. denticola* are composed of three FlaB proteins (FlaB1–FlaB3 forming the core and the FlaA protein forming the sheath).^53^ Flagella- and chemotaxis-related proteins were shown to be necessary for tissue penetration.^54^ Here we demonstrate that FlaB3 is highly expressed *in vivo* in periodontitis and therefore indeed is a virulence factor and would be a potentially useful biomarker.

### Repeat protein falo_c_1_982, biomarker from *F. alocis*

Falo_c_1_982 is a hypothetical protein of unknown function. It is 1,083 amino acids in length and contains a copper amine oxidase N-terminal domain PFAM motif (*E* value: 1.5e−21). Blastp identified the copper amine oxidase domain protein from the same species as the most similar protein sharing 32% identity (Score: 570, *E *value: 2e−81). Amine oxidase is an enzyme that converts primary amines to aldehydes with the subsequent release of ammonia and hydrogen peroxide, and allows an organism to utilise various amine substrates as a carbon and nitrogen source. This particular hypothetical protein has not yet been studied and it is a potential new virulence factor of the emerging periodontal pathogen *F. alocis*.^55^ Malodor, however, is an important symptom of periodontitis.^56^

It was proposed that key-stone pathogens might turn commensals into pathogens in the dysbiotic community.^13^ *Prevotella* species are obligatory anaerobic, Gram-negative representatives of the phylum Bacteroidetes. *Pr. intermedia* and *Pr. nigrescens* are two closely related species that can be found in subgingival plaque. *Pr. intermedia* has been associated with periodontal disease, whereas *Pr. nigrescens* was more prevalent in periodontal health. In our study, *Pr. intermedia*, but not *Pr. nigrescens*, was associated with disease. As *Pr. nigrescens* was moderately abundant in samples from both conditions, it was possible to investigate the effect of dysbiosis on its transcriptome. Indeed, we observed increased expression of virulence factors, confirming the above hypothesis that a mildly pathogenic species can switch to more pathogenic physiology in dysbiosis without a shift in abundance.

Short-chain carboxylic-acids like butyrate occur in the gingival crevices of individuals with periodontitis at millimolar concentrations and stimulate inflammation.^42^ In addition, butyrate induces the production of reactive oxygen species, inhibits growth of gingival fibroblasts^43^ and induces apoptosis and autophagic cell death in gingival epithelial cells.^41^ Butyrate was reported to be a source of energy for the host epithelial cells and has multiple roles in their physiology.^57^ Although buryrate is thought to contribute to dysbiosis in periodontitis, it may protect the gut in colorectal cancer.^58^ It has been suggested that *F. nucleatum* is a novel key-stone pathogen because expression of lysine degradation pathway leading to butyrate was observed in periodontitis patients.^17^ Here we discovered that *F. nucleatum* expressed genes for three different pathways leading to butyrate, but this was not specific for periodontitis but similar transcript abundances were also observed in health. However, in the dysbiotic community, additional species contributed to butyrate production, namely *P. gingivalis*, *P. endodontalis, T. forsythia* and *F. alocis*. In disease, the proteolytic activity (e.g., high expression of gingipains from *P. gingivalis*) of periodontal pathogens together with the autodestructive, disturbed host immune system, fuels the community with peptides and free amino acids, the substrates for butyrate production. Thus, the higher concentrations of those substrates in periodontitis may be the reason for the higher functional and taxonomic diversity of butyrate producers in disease, rather than the presence of *F. nucleatum*. According to our data, it cannot be viewed as a key-stone pathogen based on its butyrate production. *F. nucleatum* has previously been thought to be a commensal in the oral cavity important for dental plaque development owing to its strong co-aggregation potential; however, it has now been shown to be associated with a large number of different diseases, including adverse pregnancy outcomes and colorectal cancer.^59^ The virulence factor mediating all of those diverse interactions with the host is FadA, an adhesin which binds to cadherins, the conserved cell-junction molecules of epithelial cells.^59^ Unravelling the precise role of *F. nucleatum* in periodontitis, especially the processes that turns it from a commensal into a pathogen, clearly requires further studies.

## Conclusion

Our data strongly support the polymicrobial synergy and dysbiosis model for periodontitis pathogenesis. Clear shifts in the functional profiles of microbial communities from health to disease were observed that could be predicted from three highly expressed biomarker genes. Biomarkers included immunogenic surface proteins, known virulence factors and potential novel virulence traits. *Pr. nigrescens* was turned into an accessory pathogen in the dysbiotic community, and the synthesis of the key metabolite butyrate was accomplished by additional species and pathways in dysbiosis, rather than by *F. nucleatum* alone. However, it remains to be shown whether these shifts are induced by the responses of commensals to changing conditions in the gingival fluid, or by the triggering effect of key-stone pathogens or both.

## Figures and Tables

**Table 1:** Genes of *Prevotella nigrescens* differentially expressed in health and disease

*Locus tag*	*Log2 fold change*	*Log2 CPM*	P *value*	*Gene product*
pnig_c_8_1174	11.7	10.6	0.000	Hypothetical protein
pnig_c_6_998	10.2	9.1	0.000	d,d-heptose 1,7-bisphosphate phosphatase
pnig_c_6_996	10.2	9.0	0.000	Dehydrogenase
pnig_c_6_999	9.9	8.8	0.000	Hypothetical protein
pnig_c_26_1881	9.6	8.6	0.000	DNA-binding protein
pnig_c_8_1173	9.4	8.5	0.000	Hypothetical protein
pnig_c_1_3	9.1	8.2	0.003	Peptidase M26
pnig_c_6_997	8.8	7.9	0.000	Phosphoheptose isomerase
pnig_c_6_1000	8.3	7.4	0.013	Membrane protein
pnig_c_43_2127	7.9	7.3	0.044	Transposase
pnig_c_45_2145	7.6	8.6	0.000	Hypothetical protein
pnig_c_1_164	7.2	6.5	0.005	Acyl-CoA synthetase
pnig_c_1_166	6.9	6.2	0.023	Fumarate hydratase
pnig_c_53_2187	6.1	9.0	0.002	ABC transporter ATP-binding protein
pnig_c_9_1255	5.9	7.7	0.001	Porin
pnig_c_45_2144	5.5	7.3	0.006	Membrane protein
pnig_c_4_784	5.2	6.4	0.037	Endonuclease
pnig_c_6_1004	5.0	8.2	0.026	Polysaccharide biosynthesis protein
pnig_c_6_1013	4.1	8.8	0.001	NAD-utilising dehydrogenase
pnig_c_7_1093	4.0	8.9	0.000	Phosphoglucomutase
pnig_c_25_1868	4.0	13.5	0.000	
pnig_c_26_1883	3.5	9.7	0.002	Haem ABC transporter ATP-binding protein
pnig_c_25_1874	3.5	7.8	0.026	Dolichyl-phosphate beta-d-mannosyltransferase
pnig_c_6_1017	3.5	7.8	0.037	Amidase
pnig_c_7_1096	3.4	9.7	0.000	Cell division protein
pnig_c_2_296	3.4	9.5	0.001	Hypothetical protein
pnig_c_9_1256	3.3	7.7	0.047	l-asparaginase
pnig_c_4_675	3.2	8.8	0.012	Peptidase M16
pnig_c_4_751	3.1	10.4	0.000	Molecular chaperone DnaJ
pnig_c_14_1504	3.1	9.2	0.047	Surface protein
pnig_c_17_1619	3.0	9.3	0.003	Membrane protein
pnig_c_7_1098	2.9	8.4	0.044	Exodeoxyribonuclease III
pnig_c_7_1113	2.9	9.3	0.005	Cfr family radical SAM enzyme
pnig_c_11_1323	2.8	9.2	0.012	DNA topoisomerase IV subunit A
pnig_c_2_295	2.7	8.1	0.046	Hypothetical protein
pnig_c_19_1677	2.7	9.5	0.004	Cysteine desulfurase activator complex subunit SufB
pnig_c_26_1900	2.5	8.2	0.043	Hypothetical protein
pnig_c_29_1964	2.4	8.8	0.043	Multidrug transporter
pnig_c_4_746	2.4	9.6	0.044	DNA-binding protein
pnig_c_4_804	2.4	9.4	0.011	Phosphoribosylaminoimidazole-succinocarboxamide synthases
pnig_c_4_749	2.3	10.1	0.006	ABC transporter ATP-binding protein
pnig_c_4_682	2.3	10.1	0.006	Carbamoyl phosphate synthase large subunit
pnig_c_11_1334	2.3	9.6	0.023	GTPase Era
pnig_c_5_926	2.2	10.0	0.048	Imidazolonepropionase
pnig_c_30_1970	2.1	10.0	0.050	Histidyl-tRNA synthase
pnig_c_11_1333	2.1	11.1	0.023	3-oxoacyl-ACP synthase
pnig_c_24_1842	−4.4	6.6	0.044	Hemolysin haemolytic protein
pnig_c_3_525	−6.8	7.2	0.002	Hypothetical protein

Abbreviation: CPM, count of mapped reads per gene per million reads.

*P* values were corrected for false discovary rate using the method by Benjamini and Hochberg.

## Notes

Supplementary Figure S5

Supplementary Datasheet S1

Supplementary Datasheet S2

Supplementary Datasheet S3

Supplementary Datasheet S4
