# Supplementary material for: Functional biomarkers for chronic periodontitis and insights into the roles of Prevotella nigrescens and Fusobacterium nucleatum; a metatranscriptome analysis
Source: NPJ Biofilms Microbiomes. 2015 Sep 23;1:15017–. doi: 10.1038/npjbiofilms.2015.17 (PMC5515211; doi:10.1038/npjbiofilms.2015.17)
Supplement: Supplementary Information [file npjbiofilms201517-s1.doc]

Supplementary Information

**Figure S1.** **Transcriptional activity of communities based on COG analysis.** (**a**) Rarefaction curves. (**b**) Principal coordinate analysis. Bray-Curtis similarity values were calculated on standardised log-transformed abundances of reads grouped to COGs. Only COGs that at least in one sample reach the relative anundance of 0.01 % were taken into account. Communities from healthy individuals and those with periodontitis are marked in green and red respectively. TIFF format.

**Figure S2. Cluster analysis based on 40 % of reads in the periodontal metatranscriptomes.** Heat map of standardized log transformed abundance of the 5000 most highly expressed genes is shown in upper part and respective cluster analyses is shown in the bottom. Darker the color of heat map, stronger the expression. Clustering was based on Bray-Curtis similarity values that were calculated on standardized log transformed abundances of reads grouped to ORFs. Communities from healthy individuals and those with periodontitis are marked in green and red respectively. Clusters at arbitrary 30 % of similarity are highlighted in blue boxes. Three groups of transcripts with higher abundance in disease are highlighted with light red color. TIFF format.

**Figure S3**. **Cluster analysis based on 40 % to 80 % of reads in the periodontal metatranscriptomes.** Clustering was based on Bray-Curtis similarity values that were calculated on standardized log transformed abundances of reads grouped to ORFs. Communities from healthy individuals and those with periodontitis are marked in green and red respectively. **Figure S4. Separation of health- and disease- related communities using 749 gene markers and PCoA analysis. (a)** PCoA coupled with clustering analysis was based on Bray-Curtis similarity values that were calculated on standardized log transformed abundances of reads grouped to 749 gene markers. Communities from healthy individuals and those with periodontitis are marked in green and red respectively. (b) PCoA plot was overlaid with relative abundance of transcripts belonging to disease and health related oral species. Bubble size reflects % of transcripts of this species in this community. TIFF format.

**Figure S5. Composition of microbial communities from periodontal pockets as assessed by metatranscriptome and 16S amplicon profilling.** Composition of the periodontal communities in 16 individuals shown on the Class level. MET – metatranscriptome, 16S – 16S rRNA gene amplicon sequencing (V1 region, EM extraction method [Szafranski et al., 2014]). TIFF format.

**Datasheet S1. Species associated to health and periodontal disease.** Complete list of species with full taxonomy and read counts per community. For species that were associated either to health or to disease LDA score and p value is given. Excel .xlsx file.

**Datasheet S2. COGs associated to health and periodontal disease.** Complete list of COGs with full description and read counts per community. For COGs that were associated either to health or to disease LDA score and p value is given. Excel .xlsx file.

**Datasheet S3. Top marker genes of periodontal disease.** List of top 100 marker genes with species origin, LDA score, p value, read counts per community and nucleotide sequences. Excel .xlsx file.

**Datasheet S4. Genes involved in butyrate production.** List of butyrate-related genes with species and pathway origin. Read counts per 1 M reads per community and nucleotide sequence are given. Excel .xlsx file.

Table S1. Demographic and clinical characteristics of the individuals studied. Excel .xlsx file.

Table S2. Detail demographic and clinical characteristics of the individuals studied. Excel .xlsx file.

Table S3. Sequencing results. Excel .xlsx file.

Table S4. Genes of *Prevotella nigrescens* differentially expressed in health and disease. Excel .xlsx file.

Table S5. Periodontal butyrate producers – an overview. Excel .xlsx file.
