# Supplementary figures and images for: Functional biomarkers for chronic periodontitis and insights into the roles of Prevotella nigrescens and Fusobacterium nucleatum; a metatranscriptome analysis
Source: NPJ Biofilms Microbiomes. 2015 Sep 23;1:15017–. doi: 10.1038/npjbiofilms.2015.17 (PMC5515211; doi:10.1038/npjbiofilms.2015.17)

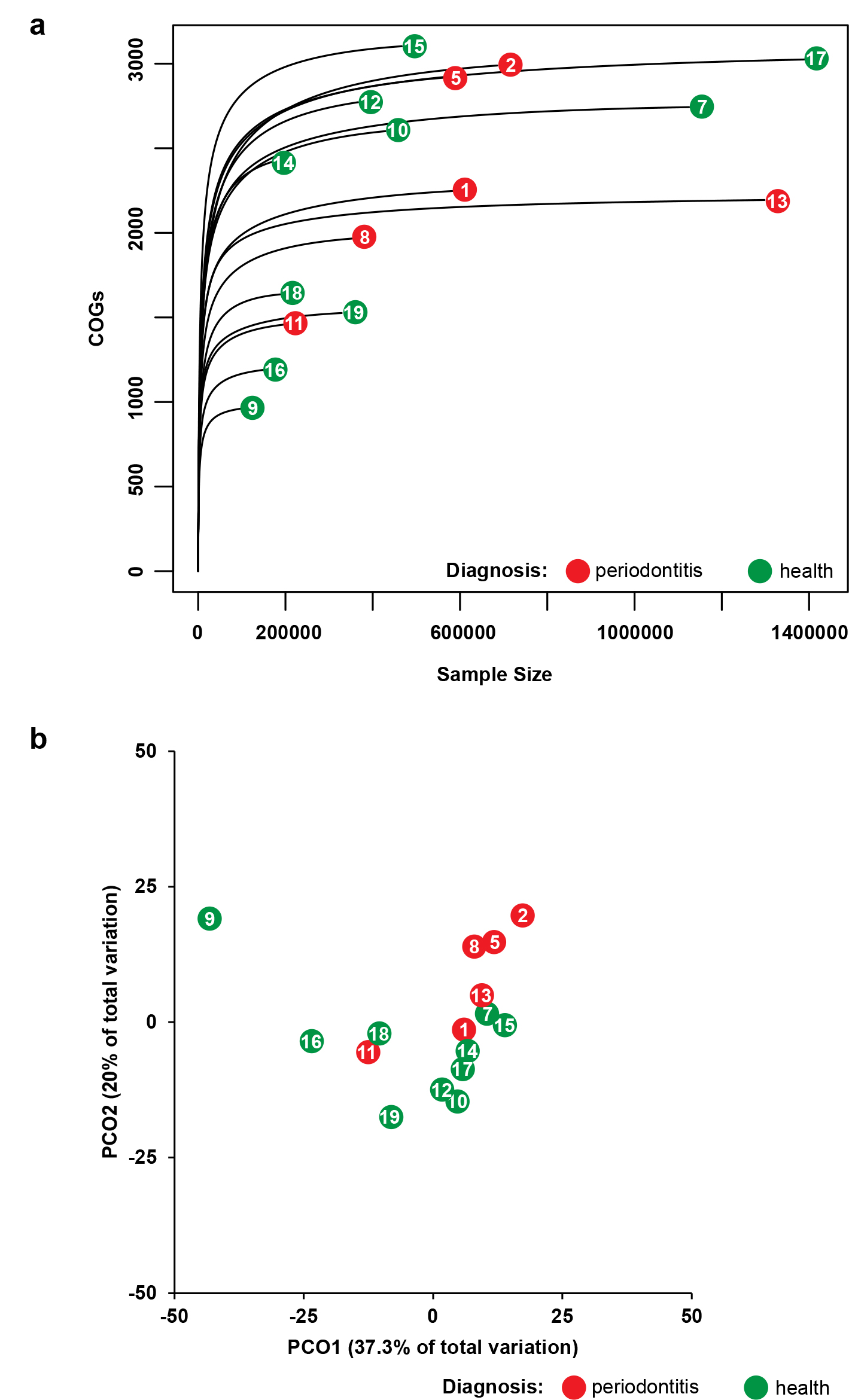

Supplement: Supplementary Figure S1 [file npjbiofilms201517-s2.jpg]

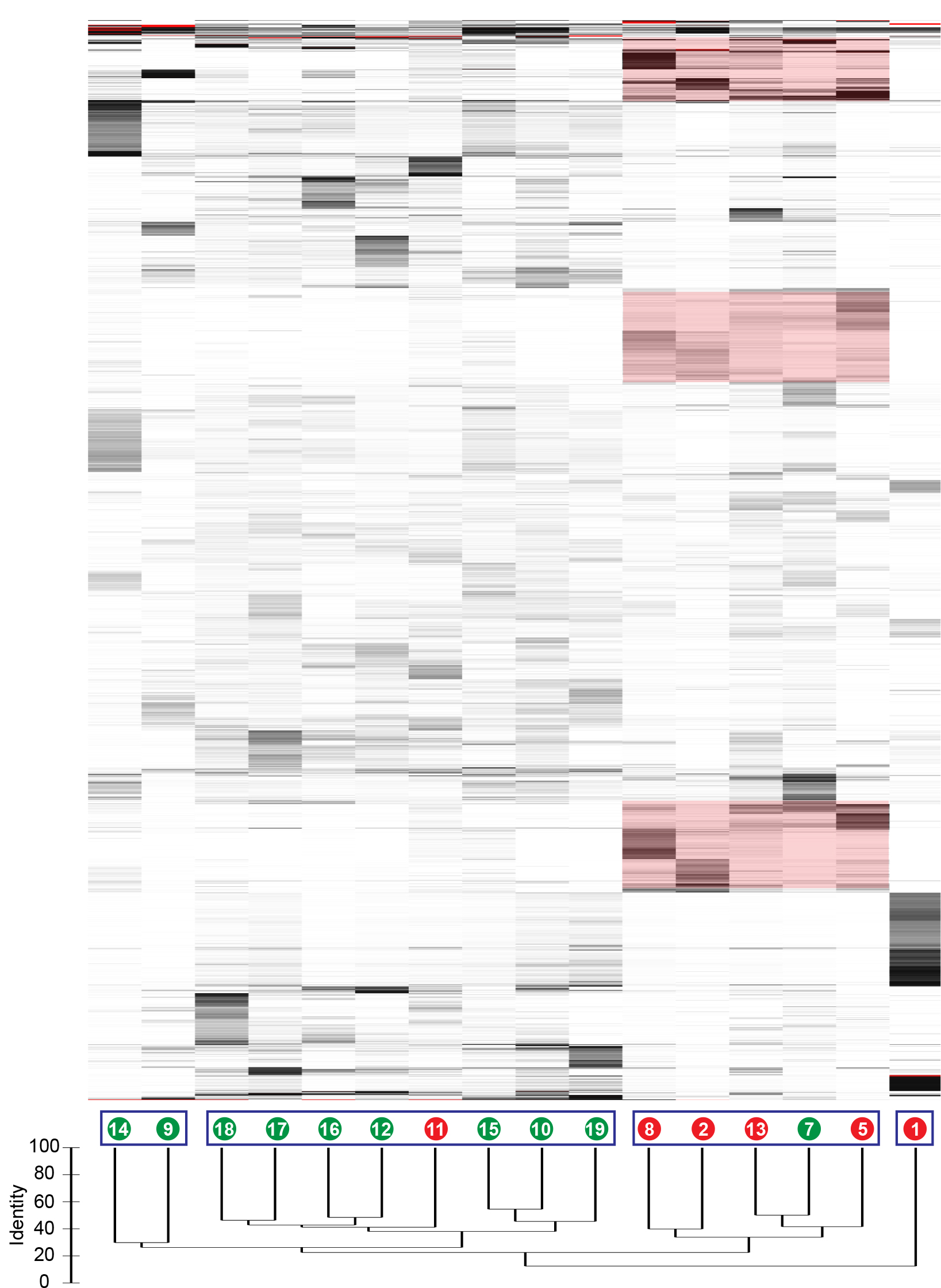

Supplement: Supplementary Figure S2 [file npjbiofilms201517-s3.jpg]

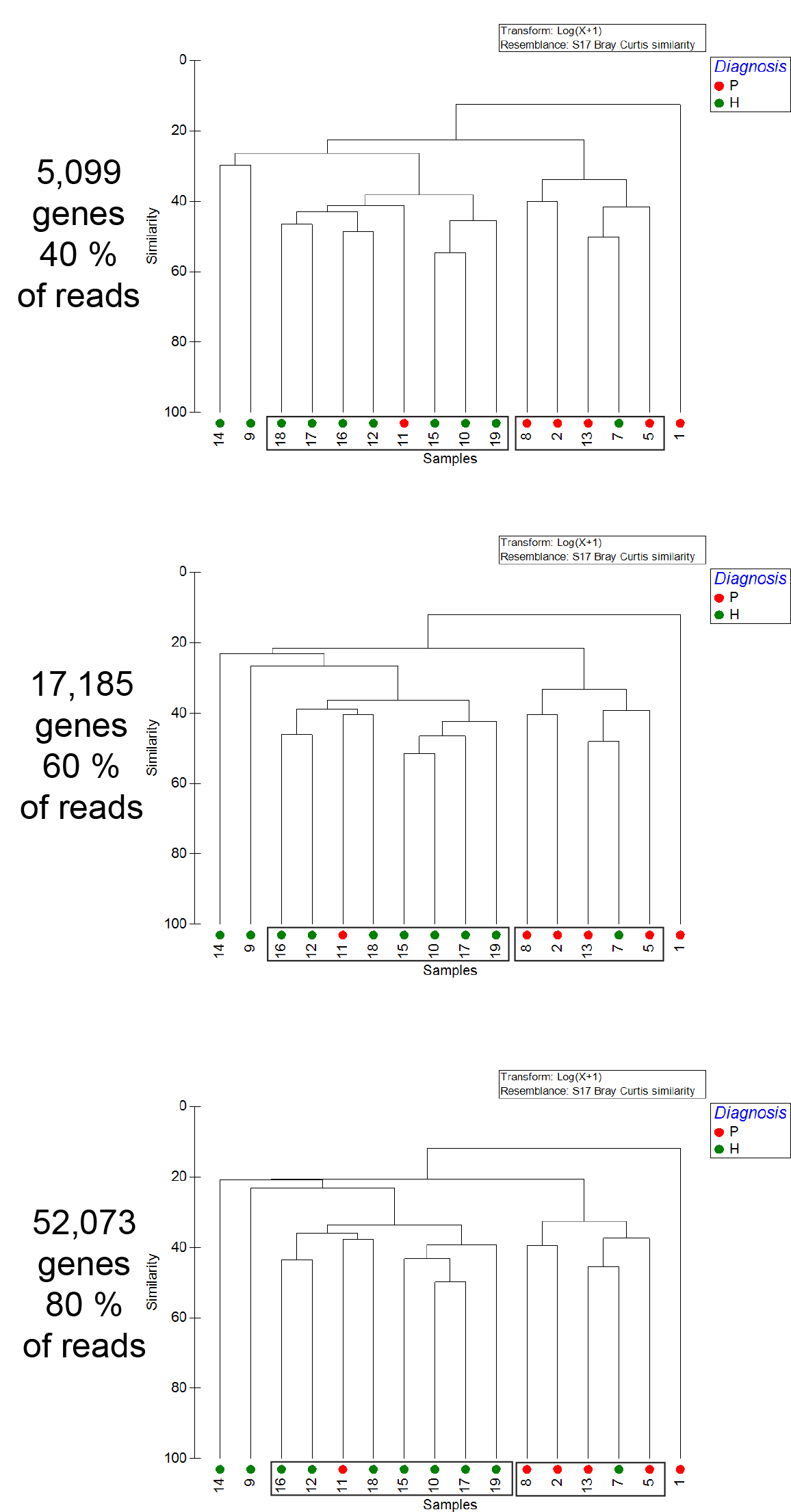

Supplement: Supplementary Figure S3 [file npjbiofilms201517-s4.jpg]

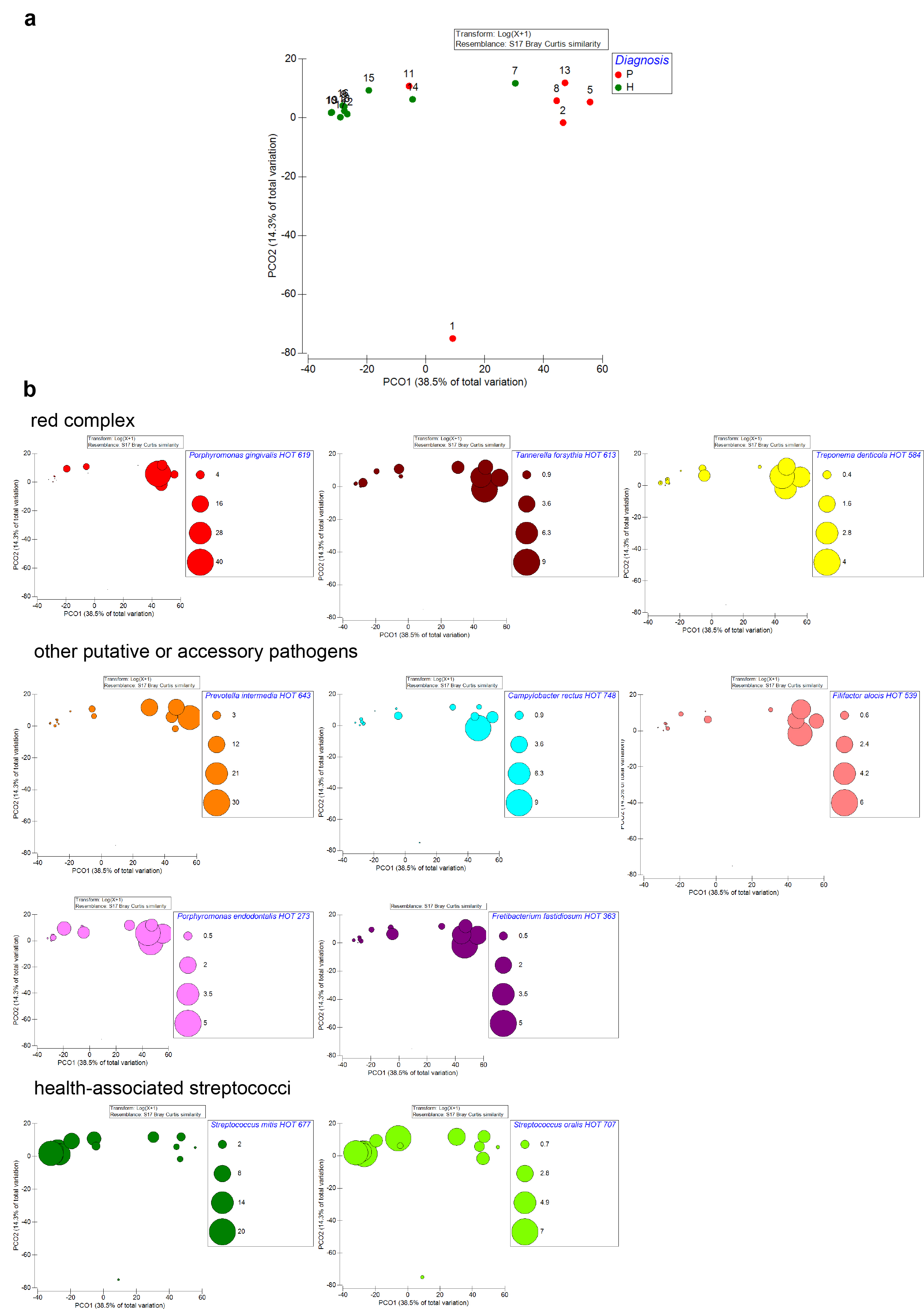

Supplement: Supplementary Figure S4 [file npjbiofilms201517-s5.jpg]

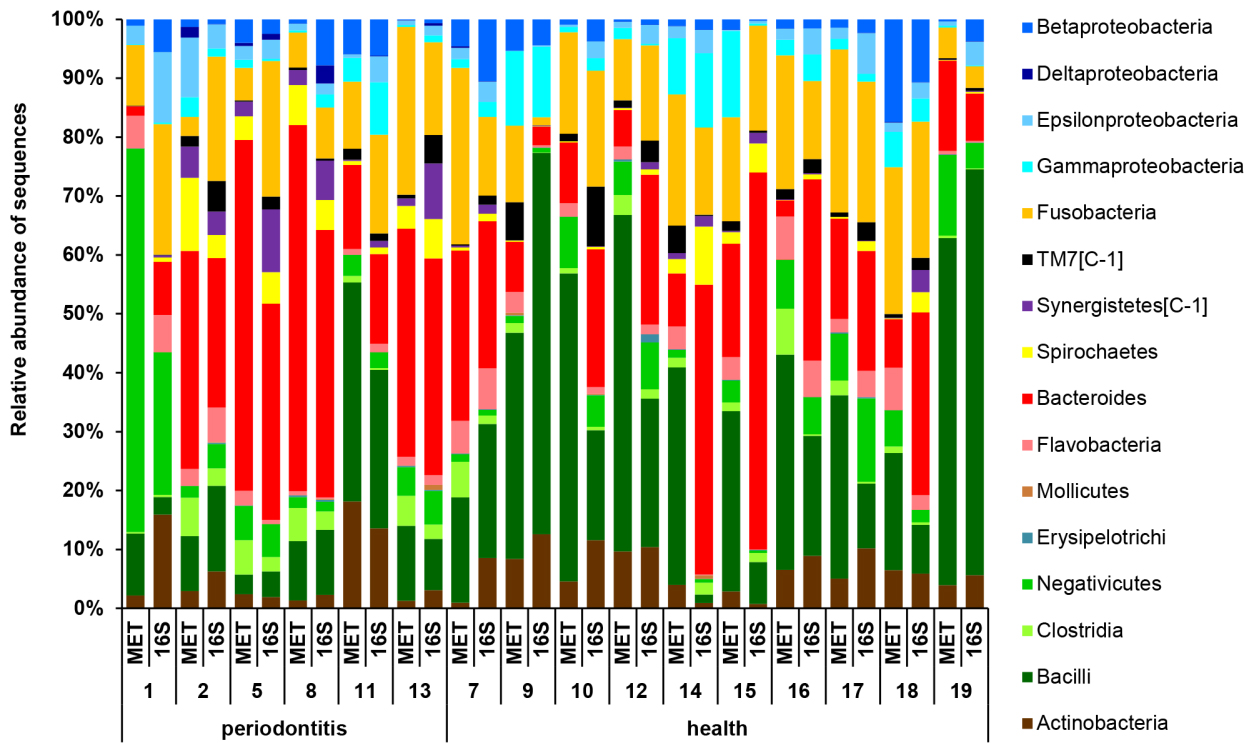

Supplement: Supplementary Figure S5 [file npjbiofilms201517-s6.jpg]
